# Supplementary material for: Analysis of PPARγ Signaling Activity in Psoriasis
Source: Int J Mol Sci. 2021 Aug 10;22(16):8603. doi: 10.3390/ijms22168603 (PMC8395241; doi:10.3390/ijms22168603)
Supplement: Supplementary file 1 [file ijms-22-08603-s001.zip › Supplemental materials_Analysis of PPARg signaling activity in psoriasis/Pathway models/Models images and html files/Model of PPARG signaling in psoriasis/112182.html]

LPS


# Small Molecule LPS

|  |  |
| --- | --- |
| URN | urn:agi-cas:93572-42-0 |
| Total Entities | 13 |
| Connectivity | 12982 |
| Name | LPS |
| Class | Endogenous compound |

---

|  |  |
| --- | --- |
| ChildConcepts | ONO-4007 |
|  | bacterial lipopolysaccharide |
|  | E5531 |
|  | Eritoran |
|  | GLA-59 |
|  | lipid A derivatives |
|  | lipid A |
|  | GLA-60 |
|  | DT-5461 |
|  | lipid X |
|  | lapretolimod |
|  | GLA-27 |
|  | glucopyranosyl lipid A |

---

|  |  |
| --- | --- |
| Pathway | Complement Alternative Pathway |
|  | Fatty Acids Block TLR Signaling in Enteroendocrine Cell |
|  | I-cell: CCK-8 Secretion and Regulation of Eating Behavior |
|  | Cortisol in Resolving Inflammation |
|  | DC-SIGN (CD209) Signaling |
|  | DCIR1 (CLEC4A) Signaling |
|  | TLR4 Signaling in Leukocytes |
|  | T-Cell Independent B-Cell Activation |
|  | Necrosis |
|  | MC1R in anti-Inflammatory Signaling |
|  | Mast-Cell Activation without Degranulation |
|  | Mast-Cell Activation without Degranulation through IL1R1 and TLR Signaling |
|  | Macrophage M1 Lineage |
|  | CD16/CD14 Proinflammatory Monocyte Activation |
|  | AHR Signaling in M1 Macrophages Function |
|  | anti-Inflammatory Function of Macrophage M2 Lineage |
|  | AXL Receptor Inhibits Macrophages and Dendritic Cells Function |
|  | Neutrophil Recruitment and Priming |
|  | Positive Acute Phase Proteins Synthesis |
|  | Dendritic Cells Function in Atherosclerosis |
|  | Toll-like Receptors (TLR) in Antiviral Innate Immune Response |
|  | Inflammatory Reaction in Acne Vulgaris |
|  | Dendritic Cells Function in Psoriasis |
|  | Plasmacytoid Dendritic Cell in Diabetes Mellitus Type 1 |
|  | TLR2 Signaling in Treg-Cell in Type 1 Diabetes (Animal Model) |
|  | Toll-like Receptors in beta-Cell |
|  | Conventional Dendritic Cell Role in Diabetes Mellitus Type 1 |
|  | Thymic Follicular Hyperplasia |
|  | Crohn's Disease |
|  | Paneth-Cell Function in Crohn's Disease |
|  | Dendritic Cell Dysfunction in Crohn's disease |
|  | Dendritic Cell Function in Ulcerative Colitis |
|  | Epithelial Cell in the Innate Immune Response in Ulcerative Colitis |
|  | Nicotine anti-Inflammatory Effect in Ulcerative Colitis |
|  | B-Cell Chronic Lymphocytic Leukemia |
|  | Club-Cell Role in Asthma |
|  | Basophil Activation in Asthma |
|  | Antigen-Presenting Cell Role in Asthma |
|  | Mucin Production in Goblet Airway Epithelial Cells |
|  | CFTR Facilitates Oxidative Stress in Airway Epithelium in CF |
|  | Airway Epithelial Cell Dysfunction |
|  | CFTR Related Ion-Channel Dysfunction in Cystic Fibrosis Airway Epithelium (Class III Mutations) |
|  | Dendritic Cell Dysfunction in Psoriatic Arthritis |
|  | TLR2 Induced Synovial Fibroblast Activation in Rheumatoid Arthritis |
|  | Toll-like Receptors (TLR) in Periodontitis |
|  | IL1 Receptor Antagonist (IL1RN) Deficiency (DIRA) |
|  | PAPA Syndrome |
|  | TNF Receptor Associated Periodic Syndrome |
|  | Mevalonate Kinase Deficiency |
|  | Blau Syndrome |
|  | IL36 Receptor Antagonist (IL1F5) Deficiency (DITRA) |
|  | Cryopyrin (NLPR3) Associated Periodic Syndromes |
|  | Familial Cold Autoinflammatory Syndrome 2 |
|  | Familial Mediterranean Fever |
|  | Microglia Activation in Parkinson's Disease |
|  | Cholesteatoma |
|  | Immune System Role in Otitis Media |
|  | Alveolar Macrophages Dysfunction in COPD |
|  | T-Cell Cytotoxic Activation |
|  | Mucin Hyperproduction in Goblet and Mucous Cells |
|  | Plasmin Role in Glomerulonephritis |
|  | Complement Activation in Glomerulonephritis |
|  | Toll-like Receptors in Pyelonephritis |
|  | NFKB Canonical Signaling Activation in Cancer |
|  | NFKB Signaling Activation by Blocking of Tumor Suppressors |
|  | NF-kB Canonical Signaling |
|  | TLR4 -> AP-1 Expression Targets |
|  | TLR4 -> AP-1/EGR1/HIF1A Expression Targets |
|  | TLR4 -> NF-kB/IRF Expression Targets |
|  | TLR4 -> IRF Signaling |
|  | TLR4/5/7/9 -> NF-kB Signaling |
|  | TLR -> AP-1 Signaling |
|  | Model of PPARG signaling in psoriasis |
|  | 1\_Inflammation in keratinocytes in the pathology of acne |
|  | 1\_The role of Toll-like receptors in pyelonephritis |
|  | 1\_Insufficient activation of immune response in the middle ear epithelium cells in otitis media |
|  | 2\_Pathogens stimulate mucins expression in the middle ear |
|  | 3\_Role of alveolar macrophages in pulmonary tissue destruction in COPD |
|  | 1\_Th2 cell response in asthma |
|  | 3\_1\_Mucus accumulation in asthma |
|  | 4\_Mucin hyperproduction in goblet and mucous cells in COPD |
|  | 2\_Paneth cell dysfunction in Crohn's disease |
|  | 1\_Defects in response to pathogens in the gut promotes inflammation in Crohn's disease |
|  | 2\_Intestine epithelial cells drive immunological responses in ulcerative colitis |
|  | 1\_Intestine epithelial cell dysfunction in ulcerative colitis |
|  | 3\_Impairment of iron release from splenic macrophages |
|  | 1\_Antigen-presenting cells promote islet dysfunction and immune system activation in T1D |
|  | Figure 4\_Diabetes mellitus Type 1: conventional dendritic cell |

---

|  |  |
| --- | --- |
| MedScan ID | 1810326 |

---

|  |  |
| --- | --- |
| Alias | lipopolysaccharide b |
|  | 93572-42-0 |
|  | lipopolysaccharides |
|  | lipopolysaccarides |
|  | lipopolysaccaride |
|  | lipopolysaccharide |
|  | LPS |

---

|  |  |
| --- | --- |
| CAS ID | 93572-42-0 |

---

|  |  |
| --- | --- |
| Reaxys ID | 20765880 |

---
